# 4,4′-Imino­dipyridinium bis­(hydrogen phthalate)

**DOI:** 10.1107/S1600536808031681

**Published:** 2008-10-22

**Authors:** David P. Martin, Robert L. LaDuca

**Affiliations:** a Lyman Briggs College, Department of Chemistry, Michigan State University, East Lansing, MI 48825, USA

## Abstract

In the title salt, C_10_H_11_N_3_^2+^·2C_8_H_5_O_4_^−^, doubly protonated 4,4′-dipyridylamine (dpa) cations participate in N—H⋯O hydrogen bonding with two hydrogen phthalate anions to form a neutral unit. Both anions contain an intramolecular O—H⋯O hydrogen bond. In the crystal structure, these units form two-dimensional layers through π–π stacking inter­actions with a centroid-to-centroid distance of 3.763 (3) Å. In turn, these layers aggregate in three dimensions by additional N—H⋯O hydrogen bonding. The assignment to the noncentrosymmetric space group *P*1 is corroborated by chemically unreasonable aromatic ring bond distances and poor *K* scale factor distributions for a disordered model in the centrosymmetric *P* 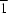 space group.

## Related literature

For the crystal structure of dpa, see: Cordes *et al.* (2006 ▶). For a chiral dpa-containing coordination polymer, see: Montney *et al.* (2007 ▶). For carboxylic acid/imine co-crystals, see: Horiuchi *et al.* (2005 ▶); Bhogala & Nangia (2003 ▶). For charge-separated hydrogen bonding, see: Steiner (2002 ▶). For the preparation of dpa, see: Zapf *et al.* (1998 ▶).
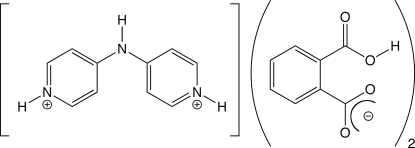


## Experimental

### 

#### Crystal data

C_10_H_11_N_3_^2+^·2C_8_H_5_O_4_^−^
*M* *_r_* = 503.46
Triclinic, 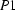

*a* = 7.858 (2) Å
*b* = 8.101 (2) Å
*c* = 9.601 (3) Å
α = 85.673 (5)°
β = 85.186 (5)°
γ = 68.834 (4)°
*V* = 567.3 (3) Å^3^
*Z* = 1
Mo *K*α radiation
μ = 0.11 mm^−1^
*T* = 293 (2) K
0.75 × 0.60 × 0.30 mm

#### Data collection

Bruker SMART 1K diffractometer
Absorption correction: multi-scan (*SADABS*; Sheldrick, 1996 ▶) *T*_min_ = 0.831, *T*_max_ = 0.967
6282 measured reflections
2513 independent reflections
2269 reflections with *I* > 2σ(*I*)
*R*_int_ = 0.014

#### Refinement

*R*[*F* ^2^ > 2σ(*F* ^2^)] = 0.060
*wR*(*F* ^2^) = 0.170
*S* = 1.06
2513 reflections
349 parameters
6 restraints
H atoms treated by a mixture of independent and constrained refinement
Δρ_max_ = 0.56 e Å^−3^
Δρ_min_ = −0.32 e Å^−3^

### 

Data collection: *SMART* (Bruker, 2001 ▶); cell refinement: *SAINT-Plus* (Bruker, 2003 ▶); data reduction: *SAINT-Plus*; program(s) used to solve structure: *SHELXS97* (Sheldrick, 2008 ▶); program(s) used to refine structure: *SHELXL97* (Sheldrick, 2008 ▶); molecular graphics: *CrystalMaker* (Palmer, 2005 ▶); software used to prepare material for publication: *SHELXL97*.

## Supplementary Material

Crystal structure: contains datablocks I, global. DOI: 10.1107/S1600536808031681/lh2702sup1.cif

Structure factors: contains datablocks I. DOI: 10.1107/S1600536808031681/lh2702Isup2.hkl

Additional supplementary materials:  crystallographic information; 3D view; checkCIF report

## Figures and Tables

**Table 1:** Hydrogen-bond geometry (Å, °)

*D*—H⋯*A*	*D*—H	H⋯*A*	*D*⋯*A*	*D*—H⋯*A*
O3—H3*A*⋯O2	1.07 (9)	1.37 (9)	2.386 (6)	155 (7)
O6—H7*A*⋯O7	1.03 (8)	1.37 (8)	2.396 (6)	172 (7)
N1—H1N⋯O1	0.86 (6)	1.90 (6)	2.757 (6)	177 (7)
N2—H2N⋯O6	0.85 (6)	2.00 (6)	2.834 (5)	167 (7)
N3—H3N⋯O8^i^	0.92 (5)	1.88 (5)	2.794 (5)	172 (5)

Symmetry code: (i) 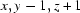.

**Fig. 1.**
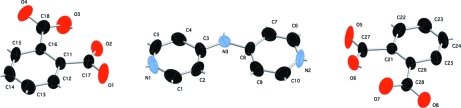
Asymmetric unit of the title compound, showing 50% probability ellipsoids and atom numbering scheme. Hydrogen atoms are represented as short gray sticks. Color codes: light-blue N, red O, black C. Hydrogen bonding is shown as dashed lines.

**Fig. 2.**
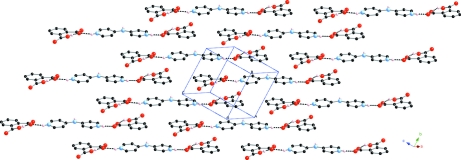
A pseudo layer in the title compound. Color codes: light-blue N, red O, black C, pink H. Hydrogen bonding is shown as dashed lines.

**Fig. 3.**
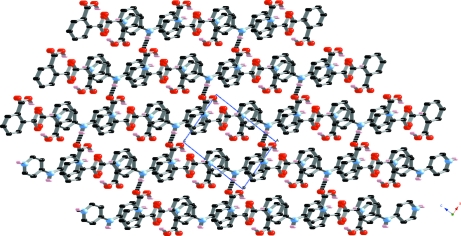
Packing diagram illustrating the stacking of layers to form the 3-D crystal structure of the title compound. Hydrogen bonding is shown as dashed lines.

## supplementary crystallographic information

### Comment

Co-crystals containing carboxylic acids and imines have exhibited enticing physical properties such as ferroelectricity (Horiuchi *et al.*, 2005). Proton transfer between carboxylic acid and pyridine components has been observed in this class of co-crystals (Bhogala & Nangia, 2003). Charge-separated hydrogen bonding interactions serve to promote the stability of these co-crystals (Steiner, 2002). Because of locked conformation between its pyridyl rings in the solid-state, crystals of pure dpa are noncentrosymmetric (Cordes *et al.*, 2006). Coordination polymers containing dpa have been observed to crystallize in noncentrosymmetric space groups (Montney *et al.*, 2007). Thus we have sought to prepare chiral dpa-containing co-crystals.

The asymmetric unit of the title salt contains a doubly protonated [H_2_dpa]^2+^ dication, and two hydrogen phthalate ([phtH]^-^) ions (Fig. 1). The similar C—O bond lengths at the carboxylate termini marked by C17 and C28 indicate the presence of delocalized π bonds. On the other hand the C—O bond distances at C18 and C27 show greater C═O and C—O single bond character.

Each [H_2_dpa]^2+^ dication is linked to [Hpht]^-^ anions on either side to form neutral [H_2_dpa][[Hpht]_2_ units, *via* N—H···O hydrogen bonding donation from both of its pyridinium termini to [Hpht]^-^ carboxylate oxygen atoms. These neutral units then engage in π–π stacking between pyridyl and benzene rings (centroid-to-centroid distance = 3.763 Å) to construct *pseudo* two-dimensional layer patterns that lie parallel to the (101) crystal planes (Fig. 2).

The [H_2_dpa][[Hpht]_2_ layers are connected into the *pseudo* three-dimensional structure of the title compound *via* N—H···O hydrogen bonding mechanisms instilled by the central amine units of the [H_2_dpa]^2+^ dications (Fig. 3). Geometric parameters for the hydrogen bonding interactions are given in Table 1.

The assignment to the noncentrosymmetric space group *P*1 is corroborated by chemically unreasonable aromatic ring bond distances (1.0 and 1.7 Å) and poor *K* scale factors distributions for a disordered model in the centrosymmetric *P*1 space group.

### Experimental

Phthalic acid was obtained commercially. 4,4'-dipyridylamine was prepared *via* a published procedure (Zapf *et al.*, 1998). A mixture of phthalic acid (96 mg, 0.58 mmol) and 4,4'-dipyridylamine (50 mg, 0.29 mmol) was placed in 10.0 g water (550 mmol) in a 25 ml beaker. The solution was then heated to boiling. Large colourless blocks of the title compound were isolated after the solution was cooled to 298K and allowed to stand undisturbed for 3 d.

### Refinement

All H atoms bound to C atoms were placed in calculated positions, with C—H = 0.95 Å and refined in riding mode with *U*_iso_ = 1.2*U*_eq_(C). All H atoms bound to O and N atoms were found *via* Fourier difference map and refined with *U*_iso_ = 1.2*U*_eq_(N) or 1.2*U*_eq_(O). The pyridinium N—H bonds were restrained with N—H = 0.89 (2) Å and the amine N—H bond was restrained with N—H = 0.85 (2) Å. The O—H distances were allowed to refine. In the absence of significant anomalous dispersion effects Friedel pairs were merged prior to the final refinement.

### Crystal data

**Table:**

C_10_H_11_N_3_^2+^·2C_8_H_5_O_4_^−^	*Z* = 1
*M**_r_* = 503.46	*F*(000) = 262
Triclinic, *P*1	*D*_x_ = 1.474 Mg m^−^^3^
Hall symbol: P 1	Mo *K*α radiation, λ = 0.71073 Å
*a* = 7.858 (2) Å	Cell parameters from 6282 reflections
*b* = 8.101 (2) Å	θ = 2.1–28.0°
*c* = 9.601 (3) Å	µ = 0.11 mm^−^^1^
α = 85.673 (5)°	*T* = 293 K
β = 85.186 (5)°	Block, colourless
γ = 68.834 (4)°	0.75 × 0.60 × 0.30 mm
*V* = 567.3 (3) Å^3^	

### Data collection

**Table:**

Bruker SMART 1K diffractometer	2513 independent reflections
Radiation source: fine-focus sealed tube	2269 reflections with *I* > 2σ(*I*)
graphite	*R*_int_ = 0.014
ω scans	θ_max_ = 28.0°, θ_min_ = 2.1°
Absorption correction: multi-scan (SADABS; Sheldrick, 1996)	*h* = −10→10
*T*_min_ = 0.831, *T*_max_ = 0.967	*k* = −10→10
6282 measured reflections	*l* = −12→12

### Refinement

**Table:**

Refinement on *F*^2^	Primary atom site location: structure-invariant direct methods
Least-squares matrix: full	Secondary atom site location: difference Fourier map
*R*[*F*^2^ > 2σ(*F*^2^)] = 0.060	Hydrogen site location: inferred from neighbouring sites
*wR*(*F*^2^) = 0.170	H atoms treated by a mixture of independent and constrained refinement
*S* = 1.06	*w* = 1/[σ^2^(*F*_o_^2^) + (0.0871*P*)^2^ + 0.2863*P*] where *P* = (*F*_o_^2^ + 2*F*_c_^2^)/3
2513 reflections	(Δ/σ)_max_ < 0.001
349 parameters	Δρ_max_ = 0.56 e Å^−^^3^
6 restraints	Δρ_min_ = −0.32 e Å^−^^3^

### Special details

**Table:**

Geometry. All e.s.d.'s (except the e.s.d. in the dihedral angle between two l.s. planes) are estimated using the full covariance matrix. The cell e.s.d.'s are taken into account individually in the estimation of e.s.d.'s in distances, angles and torsion angles; correlations between e.s.d.'s in cell parameters are only used when they are defined by crystal symmetry. An approximate (isotropic) treatment of cell e.s.d.'s is used for estimating e.s.d.'s involving l.s. planes.
Refinement. Refinement of *F*^2^ against ALL reflections. The weighted *R*-factor *wR* and goodness of fit *S* are based on *F*^2^, conventional *R*-factors *R* are based on *F*, with *F* set to zero for negative *F*^2^. The threshold expression of *F*^2^ > σ(*F*^2^) is used only for calculating *R*-factors(gt) *etc*. and is not relevant to the choice of reflections for refinement. *R*-factors based on *F*^2^ are statistically about twice as large as those based on *F*, and *R*- factors based on ALL data will be even larger.

### Fractional atomic coordinates and isotropic or equivalent isotropic displacement parameters (Å^2^)

**Table:**

	*x*	*y*	*z*	*U*_iso_*/*U*_eq_	
O1	−0.9711 (7)	0.8965 (7)	1.1049 (4)	0.0832 (14)	
O2	−0.7469 (5)	0.7612 (6)	1.2383 (5)	0.0712 (12)	
O3	−0.6750 (5)	0.5656 (7)	1.4411 (6)	0.0824 (14)	
H3A	−0.691 (11)	0.624 (10)	1.337 (9)	0.099*	
O4	−0.8021 (6)	0.3983 (6)	1.5649 (5)	0.0780 (13)	
O5	0.3019 (7)	1.2225 (8)	0.2453 (5)	0.0926 (17)	
O6	0.0684 (5)	1.3566 (6)	0.1220 (5)	0.0709 (12)	
H7A	0.043 (9)	1.430 (9)	0.028 (8)	0.085*	
O7	−0.0087 (5)	1.5473 (6)	−0.0843 (5)	0.0802 (13)	
O8	0.1036 (5)	1.7269 (5)	−0.2062 (4)	0.0654 (10)	
N1	−0.6896 (8)	0.9599 (7)	0.9469 (5)	0.0655 (14)	
H1N	−0.780 (6)	0.944 (9)	0.995 (6)	0.079*	
N2	−0.0792 (9)	1.2304 (7)	0.3688 (5)	0.0760 (18)	
H2N	−0.047 (10)	1.285 (8)	0.299 (5)	0.091*	
N3	−0.2299 (5)	0.9798 (5)	0.7185 (4)	0.0495 (8)	
H3N	−0.121 (5)	0.902 (6)	0.751 (6)	0.059*	
C1	−0.7118 (7)	1.0624 (9)	0.8305 (7)	0.0714 (17)	
H1	−0.8289	1.1282	0.8031	0.086*	
C2	−0.5601 (8)	1.0716 (7)	0.7497 (5)	0.0605 (14)	
H2	−0.5747	1.1424	0.6674	0.073*	
C3	−0.3924 (6)	0.9770 (7)	0.7916 (5)	0.0533 (12)	
C4	−0.3767 (8)	0.8754 (8)	0.9177 (6)	0.0689 (16)	
H4	−0.2625	0.8122	0.9517	0.083*	
C5	−0.5320 (10)	0.8709 (8)	0.9894 (6)	0.0713 (16)	
H5	−0.5226	0.8009	1.0718	0.086*	
C6	0.0413 (8)	1.1117 (8)	0.4402 (7)	0.0668 (15)	
H6	0.1643	1.0811	0.4115	0.080*	
C7	−0.0060 (7)	1.0321 (7)	0.5539 (6)	0.0607 (13)	
H7	0.0843	0.9471	0.6042	0.073*	
C8	−0.1878 (8)	1.0729 (6)	0.5996 (5)	0.0517 (11)	
C9	−0.3186 (7)	1.1998 (8)	0.5237 (6)	0.0618 (14)	
H9	−0.4424	1.2313	0.5500	0.074*	
C10	−0.2574 (9)	1.2797 (8)	0.4051 (6)	0.0697 (15)	
H10	−0.3414	1.3673	0.3517	0.084*	
C11	−1.0440 (6)	0.7547 (6)	1.3134 (4)	0.0392 (9)	
C12	−1.2276 (6)	0.8392 (7)	1.2857 (5)	0.0508 (11)	
H12	−1.2573	0.9150	1.2065	0.061*	
C13	−1.3670 (7)	0.8140 (8)	1.3720 (7)	0.0642 (14)	
H13	−1.4881	0.8714	1.3502	0.077*	
C14	−1.3265 (7)	0.7059 (8)	1.4877 (6)	0.0639 (15)	
H14	−1.4203	0.6922	1.5477	0.077*	
C15	−1.1462 (7)	0.6149 (7)	1.5178 (5)	0.0547 (12)	
H15	−1.1207	0.5380	1.5967	0.066*	
C16	−1.0025 (6)	0.6360 (5)	1.4328 (4)	0.0384 (9)	
C17	−0.9122 (7)	0.8067 (6)	1.2125 (5)	0.0493 (11)	
C18	−0.8166 (7)	0.5225 (7)	1.4825 (6)	0.0586 (12)	
C21	0.3638 (5)	1.3702 (5)	0.0345 (4)	0.0391 (9)	
C22	0.5473 (7)	1.2878 (7)	0.0605 (5)	0.0535 (12)	
H22	0.5797	1.2125	0.1396	0.064*	
C23	0.6836 (7)	1.3158 (8)	−0.0294 (7)	0.0640 (14)	
H23	0.8060	1.2567	−0.0120	0.077*	
C24	0.6362 (7)	1.4317 (9)	−0.1449 (6)	0.0632 (14)	
H24	0.7259	1.4535	−0.2048	0.076*	
C25	0.4570 (7)	1.5131 (7)	−0.1694 (5)	0.0539 (12)	
H25	0.4265	1.5892	−0.2483	0.065*	
C26	0.3158 (6)	1.4886 (6)	−0.0825 (4)	0.0420 (9)	
C27	0.2367 (8)	1.3142 (7)	0.1443 (5)	0.0548 (12)	
C28	0.1238 (6)	1.5967 (6)	−0.1279 (5)	0.0488 (10)	

### Atomic displacement parameters (Å^2^)

**Table:**

	*U*^11^	*U*^22^	*U*^33^	*U*^12^	*U*^13^	*U*^23^
O1	0.080 (3)	0.110 (4)	0.055 (2)	−0.037 (3)	0.000 (2)	0.034 (2)
O2	0.044 (2)	0.090 (3)	0.076 (3)	−0.0278 (18)	0.0088 (17)	0.023 (2)
O3	0.0427 (19)	0.099 (3)	0.106 (3)	−0.030 (2)	−0.022 (2)	0.032 (3)
O4	0.081 (3)	0.072 (3)	0.072 (3)	−0.020 (2)	−0.018 (2)	0.032 (2)
O5	0.083 (3)	0.134 (5)	0.058 (2)	−0.047 (3)	0.000 (2)	0.048 (3)
O6	0.046 (2)	0.083 (3)	0.079 (3)	−0.0271 (18)	0.0162 (18)	0.022 (2)
O7	0.0424 (18)	0.088 (3)	0.108 (3)	−0.0269 (18)	−0.0145 (19)	0.040 (2)
O8	0.054 (2)	0.064 (2)	0.071 (2)	−0.0164 (17)	−0.0107 (17)	0.0269 (18)
N1	0.071 (3)	0.081 (3)	0.061 (3)	−0.051 (3)	0.032 (2)	−0.020 (2)
N2	0.125 (5)	0.062 (3)	0.051 (2)	−0.054 (3)	0.037 (3)	0.000 (2)
N3	0.0458 (17)	0.056 (2)	0.0426 (18)	−0.0175 (15)	0.0023 (14)	0.0135 (15)
C1	0.040 (3)	0.084 (4)	0.082 (4)	−0.008 (3)	−0.013 (2)	−0.014 (3)
C2	0.084 (4)	0.056 (3)	0.039 (2)	−0.024 (3)	−0.011 (2)	0.018 (2)
C3	0.045 (2)	0.070 (3)	0.050 (3)	−0.028 (2)	0.0135 (19)	−0.015 (2)
C4	0.063 (3)	0.066 (3)	0.051 (3)	0.006 (2)	−0.005 (2)	0.010 (2)
C5	0.098 (5)	0.059 (3)	0.049 (3)	−0.025 (3)	0.005 (3)	0.018 (2)
C6	0.054 (3)	0.066 (3)	0.083 (4)	−0.028 (2)	0.018 (3)	−0.015 (3)
C7	0.049 (3)	0.067 (3)	0.057 (3)	−0.009 (2)	−0.007 (2)	−0.003 (2)
C8	0.076 (3)	0.043 (2)	0.038 (2)	−0.027 (2)	0.011 (2)	0.0008 (17)
C9	0.044 (2)	0.078 (4)	0.063 (3)	−0.021 (2)	0.007 (2)	−0.014 (3)
C10	0.082 (4)	0.058 (3)	0.058 (3)	−0.012 (3)	−0.018 (3)	0.014 (2)
C11	0.037 (2)	0.048 (2)	0.0359 (19)	−0.0200 (17)	−0.0010 (16)	0.0031 (16)
C12	0.040 (2)	0.059 (3)	0.051 (3)	−0.016 (2)	−0.0064 (19)	0.009 (2)
C13	0.036 (2)	0.077 (4)	0.077 (4)	−0.018 (2)	−0.004 (2)	0.002 (3)
C14	0.047 (3)	0.078 (4)	0.076 (4)	−0.037 (3)	0.015 (2)	−0.001 (3)
C15	0.060 (3)	0.065 (3)	0.048 (3)	−0.036 (2)	0.006 (2)	0.009 (2)
C16	0.041 (2)	0.041 (2)	0.038 (2)	−0.0194 (17)	−0.0055 (16)	0.0053 (16)
C17	0.054 (3)	0.055 (3)	0.041 (2)	−0.025 (2)	0.0030 (19)	0.0045 (19)
C18	0.054 (3)	0.057 (3)	0.064 (3)	−0.019 (2)	−0.012 (2)	0.009 (2)
C21	0.038 (2)	0.040 (2)	0.039 (2)	−0.0150 (16)	−0.0002 (16)	0.0020 (16)
C22	0.051 (3)	0.058 (3)	0.052 (3)	−0.020 (2)	−0.016 (2)	0.014 (2)
C23	0.034 (2)	0.077 (3)	0.083 (4)	−0.024 (2)	−0.004 (2)	0.002 (3)
C24	0.047 (3)	0.088 (4)	0.061 (3)	−0.037 (3)	0.015 (2)	0.004 (3)
C25	0.050 (3)	0.065 (3)	0.049 (3)	−0.027 (2)	−0.001 (2)	0.014 (2)
C26	0.0350 (19)	0.052 (2)	0.041 (2)	−0.0199 (17)	0.0055 (16)	−0.0010 (18)
C27	0.062 (3)	0.058 (3)	0.045 (2)	−0.026 (2)	0.015 (2)	0.004 (2)
C28	0.042 (2)	0.051 (2)	0.051 (2)	−0.0165 (18)	−0.0068 (18)	0.013 (2)

### Geometric parameters (Å, °)

**Table:**

O1—C17	1.238 (6)	C7—C8	1.385 (7)
O2—C17	1.257 (6)	C7—H7	0.9300
O2—H3A	1.37 (9)	C8—C9	1.376 (8)
O3—C18	1.305 (7)	C9—C10	1.403 (8)
O3—H3A	1.07 (9)	C9—H9	0.9300
O4—C18	1.210 (6)	C10—H10	0.9300
O5—C27	1.206 (7)	C11—C12	1.395 (6)
O6—C27	1.273 (7)	C11—C16	1.420 (6)
O6—H7A	1.03 (8)	C11—C17	1.507 (6)
O7—C28	1.273 (6)	C12—C13	1.383 (7)
O7—H7A	1.37 (8)	C12—H12	0.9300
O8—C28	1.218 (6)	C13—C14	1.347 (8)
N1—C5	1.271 (9)	C13—H13	0.9300
N1—C1	1.325 (9)	C14—C15	1.385 (8)
N1—H1N	0.86 (6)	C14—H14	0.9300
N2—C6	1.282 (9)	C15—C16	1.391 (6)
N2—C10	1.333 (9)	C15—H15	0.9300
N2—H2N	0.85 (6)	C16—C18	1.513 (7)
N3—C8	1.403 (5)	C21—C22	1.388 (6)
N3—C3	1.411 (5)	C21—C26	1.404 (6)
N3—H3N	0.92 (5)	C21—C27	1.547 (6)
C1—C2	1.389 (9)	C22—C23	1.391 (7)
C1—H1	0.9300	C22—H22	0.9300
C2—C3	1.342 (8)	C23—C24	1.382 (9)
C2—H2	0.9300	C23—H23	0.9300
C3—C4	1.400 (7)	C24—C25	1.355 (8)
C4—C5	1.361 (9)	C24—H24	0.9300
C4—H4	0.9300	C25—C26	1.394 (6)
C5—H5	0.9300	C25—H25	0.9300
C6—C7	1.321 (9)	C26—C28	1.527 (6)
C6—H6	0.9300		
			
C17—O2—H3A	114 (4)	C13—C12—C11	122.2 (4)
C18—O3—H3A	110 (4)	C13—C12—H12	118.9
C27—O6—H7A	109 (4)	C11—C12—H12	118.9
C28—O7—H7A	109 (3)	C14—C13—C12	119.7 (5)
C5—N1—C1	121.9 (5)	C14—C13—H13	120.2
C5—N1—H1N	116 (5)	C12—C13—H13	120.2
C1—N1—H1N	122 (5)	C13—C14—C15	120.4 (5)
C6—N2—C10	122.0 (5)	C13—C14—H14	119.8
C6—N2—H2N	120 (5)	C15—C14—H14	119.8
C10—N2—H2N	118 (5)	C14—C15—C16	121.4 (5)
C8—N3—C3	135.3 (4)	C14—C15—H15	119.3
C8—N3—H3N	108 (4)	C16—C15—H15	119.3
C3—N3—H3N	117 (4)	C15—C16—C11	118.6 (4)
N1—C1—C2	119.9 (5)	C15—C16—C18	113.1 (4)
N1—C1—H1	120.0	C11—C16—C18	128.3 (4)
C2—C1—H1	120.0	O1—C17—O2	121.0 (5)
C3—C2—C1	119.2 (5)	O1—C17—C11	118.5 (5)
C3—C2—H2	120.4	O2—C17—C11	120.5 (4)
C1—C2—H2	120.4	O4—C18—O3	120.8 (5)
C2—C3—C4	118.6 (5)	O4—C18—C16	119.8 (5)
C2—C3—N3	123.6 (5)	O3—C18—C16	119.3 (5)
C4—C3—N3	117.8 (5)	C22—C21—C26	118.9 (4)
C5—C4—C3	118.6 (5)	C22—C21—C27	112.6 (4)
C5—C4—H4	120.7	C26—C21—C27	128.5 (4)
C3—C4—H4	120.7	C21—C22—C23	121.3 (4)
N1—C5—C4	121.8 (5)	C21—C22—H22	119.4
N1—C5—H5	119.1	C23—C22—H22	119.4
C4—C5—H5	119.1	C24—C23—C22	119.6 (5)
N2—C6—C7	121.3 (5)	C24—C23—H23	120.2
N2—C6—H6	119.4	C22—C23—H23	120.2
C7—C6—H6	119.4	C25—C24—C23	118.9 (4)
C6—C7—C8	121.1 (5)	C25—C24—H24	120.5
C6—C7—H7	119.4	C23—C24—H24	120.5
C8—C7—H7	119.4	C24—C25—C26	123.4 (4)
C9—C8—C7	118.2 (4)	C24—C25—H25	118.3
C9—C8—N3	123.2 (5)	C26—C25—H25	118.3
C7—C8—N3	118.5 (5)	C25—C26—C21	117.8 (4)
C8—C9—C10	117.3 (5)	C25—C26—C28	114.7 (4)
C8—C9—H9	121.4	C21—C26—C28	127.5 (3)
C10—C9—H9	121.4	O5—C27—O6	121.4 (5)
N2—C10—C9	120.1 (5)	O5—C27—C21	119.0 (5)
N2—C10—H10	119.9	O6—C27—C21	119.4 (4)
C9—C10—H10	119.9	O8—C28—O7	122.3 (5)
C12—C11—C16	117.7 (4)	O8—C28—C26	118.4 (4)
C12—C11—C17	114.6 (4)	O7—C28—C26	119.3 (4)
C16—C11—C17	127.7 (4)		
			
C5—N1—C1—C2	1.5 (9)	C12—C11—C16—C18	177.2 (5)
N1—C1—C2—C3	−0.6 (9)	C17—C11—C16—C18	−4.8 (8)
C1—C2—C3—C4	−1.4 (8)	C12—C11—C17—O1	−10.2 (7)
C1—C2—C3—N3	−178.7 (5)	C16—C11—C17—O1	171.7 (5)
C8—N3—C3—C2	3.8 (8)	C12—C11—C17—O2	169.0 (5)
C8—N3—C3—C4	−173.6 (5)	C16—C11—C17—O2	−9.0 (8)
C2—C3—C4—C5	2.7 (9)	C15—C16—C18—O4	17.3 (7)
N3—C3—C4—C5	−179.9 (5)	C11—C16—C18—O4	−162.1 (5)
C1—N1—C5—C4	−0.2 (10)	C15—C16—C18—O3	−159.4 (5)
C3—C4—C5—N1	−1.9 (10)	C11—C16—C18—O3	21.3 (8)
C10—N2—C6—C7	0.1 (10)	C26—C21—C22—C23	−2.2 (8)
N2—C6—C7—C8	0.5 (9)	C27—C21—C22—C23	177.6 (5)
C6—C7—C8—C9	−0.4 (8)	C21—C22—C23—C24	1.9 (9)
C6—C7—C8—N3	178.0 (5)	C22—C23—C24—C25	−1.3 (10)
C3—N3—C8—C9	1.6 (8)	C23—C24—C25—C26	1.2 (10)
C3—N3—C8—C7	−176.7 (5)	C24—C25—C26—C21	−1.4 (8)
C7—C8—C9—C10	−0.3 (8)	C24—C25—C26—C28	178.6 (5)
N3—C8—C9—C10	−178.6 (5)	C22—C21—C26—C25	1.9 (7)
C6—N2—C10—C9	−0.8 (9)	C27—C21—C26—C25	−177.9 (5)
C8—C9—C10—N2	0.9 (9)	C22—C21—C26—C28	−178.1 (5)
C16—C11—C12—C13	1.7 (7)	C27—C21—C26—C28	2.1 (8)
C17—C11—C12—C13	−176.5 (5)	C22—C21—C27—O5	6.8 (7)
C11—C12—C13—C14	0.6 (9)	C26—C21—C27—O5	−173.4 (6)
C12—C13—C14—C15	−2.4 (9)	C22—C21—C27—O6	−168.9 (5)
C13—C14—C15—C16	1.9 (9)	C26—C21—C27—O6	10.9 (8)
C14—C15—C16—C11	0.4 (7)	C25—C26—C28—O8	−20.4 (7)
C14—C15—C16—C18	−179.0 (5)	C21—C26—C28—O8	159.6 (5)
C12—C11—C16—C15	−2.2 (6)	C25—C26—C28—O7	158.8 (5)
C17—C11—C16—C15	175.9 (5)	C21—C26—C28—O7	−21.2 (8)

### Hydrogen-bond geometry (Å, °)

**Table:**

*D*—H···*A*	*D*—H	H···*A*	*D*···*A*	*D*—H···*A*
O3—H3A···O2	1.07 (9)	1.37 (9)	2.386 (6)	155 (7)
O6—H7A···O7	1.03 (8)	1.37 (8)	2.396 (6)	172 (7)
N1—H1N···O1	0.86 (6)	1.90 (6)	2.757 (6)	177 (7)
N2—H2N···O6	0.85 (6)	2.00 (6)	2.834 (5)	167 (7)
N3—H3N···O8^i^	0.92 (5)	1.88 (5)	2.794 (5)	172 (5)

Symmetry codes: (i) *x*, *y*−1, *z*+1.
